# Fracture Risk in Type 2 Diabetes: Current Perspectives and Gender Differences

**DOI:** 10.1155/2016/1615735

**Published:** 2016-12-04

**Authors:** Giuseppina T. Russo, Annalisa Giandalia, Elisabetta L. Romeo, Morabito Nunziata, Marco Muscianisi, Maria Concetta Ruffo, Antonino Catalano, Domenico Cucinotta

**Affiliations:** Department of Clinical and Experimental Medicine, University of Messina, Messina, Italy

## Abstract

Type 2 diabetes mellitus (T2DM) is associated with an increased risk of osteoporotic fractures, resulting in disabilities and increased mortality. The pathophysiological mechanisms linking diabetes to osteoporosis have not been fully explained, but alterations in bone structure and quality are well described in diabetic subjects, likely due to a combination of different factors. Insulin deficiency and dysfunction, obesity and hyperinsulinemia, altered level of oestrogen, leptin, and adiponectin as well as diabetes-related complications, especially peripheral neuropathy, orthostatic hypotension, or reduced vision due to retinopathy may all be associated with an impairment in bone metabolism and with the increased risk of fractures. Finally, medications commonly used in the treatment of T2DM may have an impact on bone metabolism and on fracture risk, particularly in postmenopausal women. When considering the impact of hypoglycaemic drugs on bone, it is important to balance their potential direct effects on bone quality with the risk of falling-related fractures due to the associated hypoglycaemic risk. In this review, experimental and clinical evidence connecting bone metabolism and fracture risk to T2DM is discussed, with particular emphasis on hypoglycaemic treatments and gender-specific implications.

## 1. Introduction

Osteoporosis, literally “porous bone,” a disease characterized by weak bone, is a major public health problem, affecting hundreds of millions of people worldwide, predominantly postmenopausal women. In the general population, prevalence of osteoporosis and incidence of osteoporotic fractures are considerably higher in women than in men [1], because of higher bone mineral density, greater bone size, and hence a stronger bone structure in male gender [2].

Sex hormones play a central role in the physiology of bone by direct and indirect mechanisms and the abrupt loss of estrogens at menopause onset is considered the major reason for primary osteoporosis in women; conversely, a dramatic loss of androgens with aging is lacking in men [2]. The main clinical consequences of the disease are bone fractures, especially at the hip and spine, which may be associated with serious complications such as substantial pain, disability, and even death. Dual energy X-ray absorptiometry (DXA) represents the gold standard for the diagnosis of osteoporosis [3]. According to the World Health Organization, among postmenopausal women and men 50 years old and older, diagnosis is based on *T*-score (normal values, >1.0; osteopenia, –1 to –2.5; and osteoporosis, <2.5 SD) [4].

The report “Osteoporosis in the European Union: Medical Management, Epidemiology and Economic Burden” describes the burden of osteoporosis in the EU in 2010. Twenty-two million women and 5.5 million men were estimated to have osteoporosis, with 3.5 million new fragility fractures, including 620,000 hip fractures, 520,000 vertebral fractures, 560,000 forearm fractures, and 1,800,000 fractures in other sites [1]. As a consequence, osteoporosis imposes a significant economic burden that goes beyond the medical one. Thus, the economic burden of incident and prior fragility fractures was estimated at €37 billion and these costs are expected to increase by 25% in 2025 [5].

The primary aim of pharmacological therapy is to reduce fractures risk. Although a range of medications have become available for treatment and prevention of osteoporosis during the past 4 decades, the majority of individuals who have experienced an osteoporosis-related fracture or who are at high risk of fracture are untreated and the number of patients on treatment is declining. Finally, longevity has resulted in an increasing number of subjects at higher risk for osteoporotic fractures and its related comorbidities [1].

Type 2 diabetes (T2DM) prevalence is increasing worldwide, and this increase affects especially the elderly population. As a consequence, the number of T2DM patients with osteoporosis will further increase, posing elderly patients in a vicious circle of disability due to both increased incidence of fractures and micro- and macrovascular diabetes-related complications.

Osteoporosis is a gender-related disease and postmenopausal women with diabetes, who are a particularly fragile population because of the higher cardiovascular disease-related risk [6, 7], are those at significantly higher risk for osteoporosis and its complications [8].

This review will update current knowledge on bone metabolism and fracture risk associated with T2DM, particularly focusing on potential gender differences.

## 2. Fracture Risk in Type 2 Diabetes

Although osteoporosis and T2DM seem to be unrelated from a pathophysiological standpoint, a number of epidemiological studies have demonstrated an increased fracture risk among patients with T2DM.

The first studies on the association between T2DM and fractures risk produced controversial results. The Rotterdam study on 5931 subjects (2481 men and 3450 women aged ≥ 55 years, of whom 578 were T2DM) showed a greater bone mineral density (BMD) as evaluated by the DXA in T2DM patients than in subjects with normal glucose homeostasis, and a lower frequency of nonvertebral fractures among T2DM women in the 5 years prior to inclusion in the study [9]. Thus, T2DM women reported having had fewer fractures in the 5 preceding years than women without this condition (adjusted odds ratio, 0.63; 95% CI, 0.44 to 0.90), whereas the frequency of fractures in men was similar for those with and without T2DM (adjusted odds ratio, 0.96; CI, 0.60 to 1.52) [9].

Subsequent studies, on the contrary, reported an increased incidence of fractures in T2DM. In 2005, the same group of the Rotterdam study [10] reexamined the data of 6655 men and women, making a further distinction among T2DM patients who were already treated or newly diagnosed. Although people with T2DM, men and women combined, had a higher BMD, they had an increased risk of nonvertebral fractures compared with subjects without T2DM. When data were stratified by gender, a comparable trend was observed. This increase was confined to T2DM subjects already on therapy, while those with a recent diagnosis had no increase in the risk of fractures and those with glucose intolerance had even a reduction of 20 to 40% of the risk of fractures [10]. In the Health, Aging and Body Composition Study [11] (2979 subjects, 19% with T2DM, 6% with glucose intolerance) a high risk of fractures was observed (64%, relative risk RR: 1.64) among diabetic patients, while glucose intolerance was not significantly related with the risk of fractures (RR 1.34). Diabetic patients with fractures had a higher prevalence of peripheral neuropathy, TIA/stroke, and falls compared to patients with diabetes without fractures. The most common sites of fractures among diabetic patients and people with impaired glucose tolerance were the forearm (21%), vertebrae (18%), hip (18%), tibia/fibula/ankle (10%), and foot (9%). Koh et al. [12] described the association between diabetes and fracture risk in a population-based prospective cohort study of 63237 Chinese men and women who were followed up for a mean duration of 12 years. After adjustment for other major risk factors, including self-reported calcium consumption, the risk of hip fracture was significantly increased among people with diabetes compared to people without diabetes (RR, 1.98; 95% CI, 1.71–2.29), this risk increased with duration of diabetes, and the risk estimates were similar between men and women, as well as between lean and obese individuals. Also the case-control study in a Danish national database on over 120000 subjects [13] and the retrospective cohort analysis conducted on 197412 residents aged > 66 years in Canada [14] confirmed the higher fracture risk in T2DM subjects, irrespective of the use of antidiabetic agents or diabetes-related complications.

More recent large studies confirmed the tendency toward an increased fracture risk among T2DM patients, especially women. In a prospective study of 32,089 postmenopausal women, the Iowa Women's Health Study, the risk of hip fractures was 1.7 times higher among self-reported T2DM patients, after adjusting for several risk factors [15]. In the Study of Osteoporotic Fractures (SOF) T2DM participants (*n* = 657) had a 22% higher risk of nonspine fractures than those without T2DM (*n* = 8997) [16]. The Women's Health Initiative Observational Study, including 93000 postmenopausal women, of whom 5285 subjects had T2DM, prospectively followed up for 7 years, showed a significantly higher risk of fracture in several sites in T2DM women, after controlling for multiple risk factors, including a previous history of falls [17]. Similar data were observed in the longer follow-up (22 years) of the Nurses' Health Study, showing an increased risk both in type 1 diabetes mellitus (T1DM) (*n* = 292) and T2DM (*n* = 8348) [RR: 2.2 (95% CI, 1.87–2.7); after adjustment for other risk factors] [18].

Overall, fracture risk is almost two times higher in T2DM subjects compared with nondiabetic ones, both in men and in women, although most of the studies are conducted on postmenopausal women and typically considered those at higher osteoporosis risk. Epidemiological studies that specifically compared fracture risk in T2DM men versus T2DM women are not available to date, and the few indirect comparisons do not report significant gender differences. Furthermore, the dependence of fracture risk upon diabetes duration and its long-term complications is still controversial.

## 3. Potential Pathophysiological Basis of the Increased Fracture Risk in Type 2 Diabetes

The possible influence of T2DM on fracture risk has been explained with different mechanisms that may be specifically linked to diabetes, its complications, and/or management. Among these factors, current therapies, peripheral neuropathy, reduced vision (caused by peripheral retinopathy and cataracts), hypoglycaemia, decreased muscle performance, diabetic foot, orthostatic hypotension, polyuria and nocturia, causing falls especially at night, reduction of reflexes, stroke, and cognitive impairment may all play an important role [19, 20]. Moreover, diabetes is associated with a delay in the wound healing [21], altered biochemical properties, and a reduction of cell proliferation and of collagen content in bone callus [22].

Paradoxically, patients with T2DM often have a normal or high BMD, probably associated with obesity as well as with hyperinsulinemia, altered level of estrogen, and/or adipokines. Despite this evidence, the risk of fractures in T2DM patients is higher and this finding could be related to the altered bone quality that does not emerge from measurements of BMD. Thus, diabetes can interfere with bone tissue causing impaired bone quality through different mechanisms [23], including glycosuria which may result in hypercalciuria and loss of bone mass; accumulation of the advanced glycosylation end products (AGEs) in the collagen fibers with alteration of the structure and of the strength of the bone; low levels of insulin like growth factors-I (IGF-I) considered as a bone anabolic factor; alteration in plasma insulin levels; impaired kidney function; bone microangiopathy with reduction of vascular flow and increased bone fragility and chronic inflammation with increase of cytokines that can accelerate the bone remodeling and loss of BMD. Further metabolic alterations could contribute to the increase of fracture risk in T2DM. Among these, high levels of homocysteine (tHcy) have been proposed as a risk factor for bone alteration and fracture risk also in postmenopausal women [24], and it has been demonstrated that tHcy levels increase after menopause in T2DM women as in not diabetic ones [25]. High tHcy levels may also indirectly influence fracture risk in T2DM, by increasing the incidence of micro- and macroangiopathy, although these associations remain to date still controversial [26, 27].

As for diabetes-specific mechanisms, several data indicate an effect of AGEs on collagen and bone cells. It was demonstrated that AGEs accumulating in the collagen stimulate IL-6 production in human bone cells [28], inhibit the phenotypic expression of osteoblasts and their differentiation and mineralization, inhibit type 1 collagen synthesis, and favor the formation of weak bridges between the collagen fibers resulting in the reduction of bone strength and in the increase of bone resorption induced by osteoclasts [29, 30]. These observations are corroborated by the presence of AGEs receptors (RAGEs) on bone cells [31]. A negative correlation between the serum levels of osteocalcin, a protein secreted by osteoblasts, and plasma glucose, fat mass and atherosclerosis in patients with T2DM was also observed [22]. Osteocalcin's function is peculiar since this protein exerts its effects not only on bone, but also on glucose and fat metabolism, working like a hormone able to regulate gene expression of *β*-pancreatic cells and of adipocytes and preventing the development of metabolic disease, obesity, and hyperglycaemia [32]. Furthermore, adiponectin, an adipose-tissue derived hormone, was shown to induce the proliferation, differentiation, and mineralization of osteoblasts [33].

Also altered IGF-1 levels have been associated with bone abnormalities. IGF-I is also synthesized by osteoblasts and it is a regulator of bone cells metabolism [34]. Several studies showed a reduced IGF-1 activity when glucose and AGEs levels are high, suggesting an osteoblastic resistance to IGF-1 effects [35, 36]. Kanazawa et al. showed an inverse relationship between IGF-I levels and vertebral fractures in postmenopausal T2DM women, suggesting a protective role of IGF-1 related to its effects on bone quality [37].

Also chronic inflammation may be a link between bone abnormalities and fracture risk in diabetes [38]. Inflammation induced by obesity inhibits the synthesis and secretion of adiponectin from adipose tissue, which may in turn have consequences on bone metabolism.

Among the mechanisms linking bone metabolism to T2DM, vitamin D deficiency has been extensively treated by other authors [39] and certainly merits a specific dissertation that goes beyond the aims of this review.

The relationship between the metabolic control in T2DM and bone metabolism has been the topic of numerous experimental and epidemiological studies. The results are often controversial, being influenced by the number of patients included, the study design, or the measures of glucose control. Hyperglycaemia has been shown to have a negative effect on the expression and secretion of osteocalcin by osteoblasts, and hypoglycaemic therapies can improve the levels of osteocalcin in patients with T2DM [40]. However, the Health, Aging and Body Composition Study [11] showed that T2DM patients with and without fractures had similar glycaemic control. Strotmeyer et al. [41] found no significant correlation between HbA1c levels, BMD, and bone volume, but they showed a negative correlation between the duration of the disease and hip BMD, with the hip BMD mean values progressively decreasing from the cases with a recent diagnosis of diabetes, to those with more than 20 years of diabetes, every 5–10 years' intervals. On the other hand, other studies observed an improvement and a stabilization in BMD in patients with T1DM in good metabolic control [42, 43]. Another study, aimed at evaluating the causes of low bone quality in diabetic subjects, identified low PTH levels accompanied by low bone formation as a potential contributor to the high vertebral fracture risk independently of bone mineral density risk in T2DM postmenopausal women [44].

All these experimental evidences support the pathogenic role of insulin-resistance, chronic inflammation, and long-term diabetes-specific factors, such as the formation of AGEs on the alterations of bone structure, that are at the basis of the increased fracture risk in T2DM patients. More controversial is the role of glucose control on bone mass measures, such as BMD.

To date, no gender-specific differences have been reported in these pathogenic mechanisms, although an already fragile bone such as that observed in postmenopausal women due to hormonal loss may certainly play a role in accelerating bone structure disruption.

## 4. Bone Mass versus Bone Quality in Type 2 Diabetes

The first data evaluating osteoporosis in T2DM showed high BMD values when compared to nondiabetic controls [22]. However, a subsequent meta-analysis showed that patients with T2DM had a higher risk of fractures in spite of this higher BMD, highlighting the discrepancies between BMD and fracture risk and suggesting that measuring BMD may not reflect bone fragility of these patients [45].

Gorman et al. [46] assessed bone status in older adults with and without T2DM through a literature review. Some of these studies were not limited to the use of DXA but used more recent techniques, such as quantitative computed tomography (QCT) [47], the peripheral quantitative computed tomography (pQCT) [48], and quantitative ultrasound (QUS) [49, 50], that allow distinguishing the bone compartments (cortical and trabecular), assessing bone quality (microarchitecture and geometry), and estimating bone strength. Results obtained with the use of DXA were consistent with previous studies, showing an equal or higher BMD among older adults with diabetes compared with controls [31]; conversely, those studies using the QCT and pQCT [47–50] suggested the presence of profound changes in bone geometry in diabetic subjects, potentially explaining the increased risk of fractures observed in these patients. In addition, phalangeal quantitative ultrasound (QUS) has been increasingly used for its ease of use and because it may be more helpful than DXA in detecting bone deficits, also in diabetic subjects [49, 51]. Recently, the use of DXA based trabecular bone score has been proposed as a new complementary approach to ameliorate fracture risk prediction in T2DM [52].

All these evidences suggest that the DXA alone is not able to predict the risk of fractures in older adults with diabetes, where bone health may depend upon too many factors, including BMI. At this regard, Shan et al. [53] observed that T2DM patients with a greater BMD were those with greater BMI, suggesting that BMD measures may be overestimated in obese subjects.

Since the measurement of BMD is not capable of predicting the risk of fractures among people with T2DM, it is necessary to have valid instruments to determine, in the clinical practice, not only fracture risk but also the most appropriate time to start a proper therapy. In a large study of postmenopausal women there was a greater chance of new fracture (vertebral or even) among those who had already had a vertebral fracture; the authors highlighted the possible use of prior vertebral fractures as an indicator of bone quality in these patients [54]. Furthermore, Yamamoto et al. observed radiographic vertebral fractures in 38% of T2DM males and 31% of T2DM females, with a 16% of the subjects having a personal history of previous fractures, and concluded that simple procedures such as medical history and X-ray can be used in clinical practice for the assessment of fracture risk in the diabetic population [55].

Among the different tools to assess fracture risk, the WHO fracture risk assessment (FRAX) is a computer-based algorithm (http://www.shef.ac.uk/FRAX/) primarily intended for use in primary care [56, 57]. FRAX calculates fracture probability from easily obtained clinical risk factors: age, sex, BMI, prolonged use of glucocorticoids, current smoking, alcohol intake of three or more units per day, a parental history of hip fracture, secondary osteoporosis, rheumatoid arthritis, prior fragility fracture, and (optionally) femoral neck BMD or *T*-score. The output, which estimates probabilities for major osteoporotic fracture (hip, clinical spine, humerus, or forearm) and hip fracture over 10 years, has been shown to improve fracture prediction over *T*-score alone [58]. Although T2DM is not a primary entry variable in the current FRAX construction, T1D is considered in FRAX as one of the secondary causes of osteoporosis, increasing the calculated fracture probability when BMD is not known. Furthermore, two recent reports have shown that, for a given FRAX probability or *T*-score and age, the risk of fracture among individuals with diabetes is higher than the risk in nondiabetics [59, 60]. In another study, mean FRAX hip fracture and FRAX major osteoporotic fracture were significantly higher in the T2DM cohort as compared to the healthy age-matched males [61, 62].

To date few studies specifically addressed potential gender differences in BMD measures in T2DM. One of the first studies by Barrett-Connor and Holbrook [63] evaluated the association of T2DM with BMD in men and women, separately. Men with diabetes had BMD levels similar to those with normal glucose tolerance, whereas women with diabetes had significantly higher BMD levels at all sites than control women, and these differences were unexplained by several potential confounders such as age, obesity, cigarette smoking, alcohol intake, regular physical activity, and the use of diuretics and estrogen. The authors related these diabetes-related differences to the greater androgenicity reported in hyperinsulinemic T2DM women. These results were confirmed in another study showing that diabetic men had a BMD similar to that of the control group, whereas diabetic women had a higher BMD than controls, showing a positive relationship between BMD and triglycerides and a negative relationship with HDL-C only in women [64]. A recent study evaluating bone metabolism by measuring markers of bone turnover and BMD, taking into account the presence of diabetic polyneuropathy (PNP), showed that male diabetic patients with PNP had a higher rate of bone turnover than men without PNP, indicating neuropathy as a potential risk factor for osteoporosis and fracture risk, beyond the risk associated with falls [65]. Finally, in a large population of men and women undergoing hip DXA, including 2929 women and 460 men with known diabetes, women had significantly lower mean spine-hip thickness differences than men (3.3 ± 1.4 cm versus 5.4 ± 1.7 cm; *p* < 0.001), which persisted after adjustment for sex-specific differences of age and BMI. Logistic regression showed that a greater spine-hip thickness difference was significantly associated with higher likelihood of having diabetes even after adjustment for age and BMI, and this effect was stronger among women than among men [66].

Despite the higher BMD found in T2DM subjects, fracture risk remains high in these patients, suggesting that BMD alone does not reflect the profound rearrangement of bone structure associated with metabolic disease. This knowledge has led some authors to introduce the term of “diabetic osteodystrophy” [67] and to search other methods to assess bone quality in T2DM patients. When specifically addressing gender differences in this issue, the few available studies that have evaluated separately men and women with diabetes relied on BMD measures, and most of them indicated high BMD values in T2DM women but not in men, when compared to control population. Studies using other markers of bone quality in T2DM are urgently needed to establish whether the higher fracture risk observed in T2DM is only related to the risk of falls associated to diabetes management and/or long-term complications, or more likely to specific alterations in bone metabolism, and finally whether all these factors differently affect T2DM men and women.

## 5. Effects of Hypoglycaemic Drugs on Bone Metabolism and Fracture Risk

Hypoglycaemic drugs may also affect bone metabolism and influence fracture risk in many ways, including the increase of bone turnover and skeletal fragility, the loss of the anabolic effects of insulin in insulin-resistant states, and by augmenting the risk of falling due to hypoglycaemic episodes [68].

### 5.1. Insulin Therapy

It is well known that insulin exerts anabolic effects on bone, which include the regulation of bone cells proliferation and apoptosis, and the synthesis of collagen [22], probably through direct receptor-mediated effects since insulin receptors were identified on osteoblasts and their precursors. However, insulin treatment is associated with a higher rate of falls and with an increased fracture risk, both in men and in women [69, 70]. This higher risk could also reflect the fact that insulin therapy is usually employed in T2DM patients with a longer diabetes duration, when multiple chronic complications and comorbidities are common [71]. Moreover, hypoglycaemic episodes are a major complication of the treatment with insulin and may imply a high rate of falls in insulin-treated subjects. Accordingly, Kennedy et al. observed that insulin-treated subjects were more likely to fall and to have bone fracture as a consequence of the fall, during a hypoglycaemic episode as compared to non-insulin-treated patients [72].

### 5.2. Metformin

Available data suggest that metformin has positive effects on bone metabolism.* In vitro *and animal studies indicated that metformin inhibits adipocyte differentiation and stimulates osteoblasts' differentiation, through the inhibition of PPAR gamma [73] and the transactivation of osteoblast-specific Runx2 transcription factor [74]. Moreover, the drug also stimulates osteoblastic expression of osteoprotegerin and depresses that of RANKL, which in turn inhibits osteoclast function and bone loss [75]. Notably, RANKL has been recently proposed as a predictor of incident T2DM, and its blockade resulted in significant improvements of glucose tolerance [76, 77].

Metformin could also play a protective role on osteoblasts, by limiting the detrimental effects of AGEs. These mechanisms could account for the reduced fracture risk observed in metformin-treated patients, despite the decrease of insulin plasma levels [78]. When considering fracture risk in these patients it is however important to keep in mind that metformin is usually prescribed in younger subjects with lower complications and comorbidities rates, who also may present a lower risk of bone fracture.

Furthermore, the potential protective effect of metformin on bone and fractures risk, suggested in animal models [73, 74], was not confirmed in clinical studies [78].

For this and other reasons, including the paucity of clinical data, the protective role of metformin on bone is still debated [70].

### 5.3. Sulfonylureas

Sparse results suggest a protective effect of sulfonylureas on fracture risk [79]. However, hypoglycaemia is a common adverse effect of the treatment with this class of drugs. A recent review evaluating the risk of fall-related fracture in T2DM subjects on sulfonylureas concluded that available studies suffer methodological limitations and may have underestimated the risk [80, 81]. Further studies are needed to define the effect of these drugs on falls and fractures, although the overall beneficial effects of this class of drugs are currently debated [34, 82].

### 5.4. Thiazolidinediones

Thiazolidinediones have been shown to exert detrimental effects on the skeleton [83]. These insulin-sensitizing agents significantly increase the incidence of bone fracture, at least in T2DM postmenopausal women, whereas less conclusive results were obtained in men. The ADOPT (A Diabetes Outcome Progression Testing) study, comparing rosiglitazone with metformin and glyburide monotherapy in patients with recently diagnosed T2DM, showed a two-fold increased incidence of bone fractures in women treated with rosiglitazone in comparison with other treatment groups [84]. Interestingly, no difference in bone fractures was found in men. The increased bone fractures risk in rosiglitazone-treated group was observed both in postmenopausal and in premenopausal women and did not appear to be modified by estrogen use. The site of fractures was atypical compared with those related to postmenopausal osteoporosis (hip and spine), with more frequent fractures observed in upper and lower limbs (proximal humerus RR > 8; hand RR = 2.6; foot RR = 3.3). However these data may be related to the age of the population (<65 years) in which the rate of hip and spine fractures is relatively low.* Post-hoc* analyses showed that the increase in fracture risk was evident after about one year of treatment [85]. An increased fracture risk was observed also in women, but not in men, treated with pioglitazone, as reported in a letter to health care providers by Takeda Pharmaceuticals, IL, USA, the manufacturer of pioglitazone [86]. Clinical trials testing the short-term effects of rosiglitazone and pioglitazone on markers of bone formation in different populations suggest that, in women, thiazolidinediones cause a more rapid bone loss. In particular, modifications in bone turnover markers indicate a pattern of reduced bone formation without a change in resorption [87–89]. Thiazolidinediones ameliorate insulin sensitivity of muscle and adipose tissue, by acting as agonists of peroxisome proliferator-activated receptor-gamma (PPAR gamma). Via the same mechanism, however, they promote an imbalance in bone remodeling and changes in bone marrow structure and function. The bone loss may result from the preferential differentiation of mesenchymal stem cells into adipocytes rather than osteoblasts, and the increase of osteoclast activity [90]. These data are consistent with two observational clinical trials demonstrating bone loss in T2DM subjects treated with rosiglitazone and pioglitazone, an effect that seems to correlate with the duration of treatment [91, 92].

### 5.5. Incretin-Based Therapies

In addition to their beneficial effects on glucose metabolism and cardiovascular risk factors [93], several data indicate that incretin-based drugs, that is, glucagon like peptide-1 receptor agonists (GLP-1-RAs) and inhibitors of the dipeptidyl peptidase-4 enzyme (DPP-4), may positively affect bone metabolism.

Bone cells, including osteoblasts and osteoclasts, express receptors for GLP-1 and data from animal studies suggest that incretins play a regulatory role in bone turnover, in response to ingestion of nutrients. This regulatory activity results in increased bone formation and reduced bone resorption in times of energy sufficiency. In particular, GLP-1 inhibits bone resorption through a calcitonin-dependent way [94]. Also the favorable effects of GLP-1RAs on body weight may influence bone metabolism; however exenatide twice daily did not affect BMD and markers of bone homeostasis in T2DM subjects, as compared to insulin glargine, despite body weight reduction [95].

Notably, incretin-based therapy is also associated with a low hypoglycaemic risk, and it may potentially reduce the fall-related fractures in T2DM subjects. However, clinical data available to date are too limited and not conclusive yet. Recently, a meta-analysis investigated the association of treatment with the GLP-1RAs exenatide and liraglutide with bone fractures incidence in T2DM subjects, showing no effects on fracture risk, as compared to placebo or other antidiabetic drugs (glimepiride, sitagliptin, and insulin) [96]. Conversely, another recent meta-analysis showed an increased risk of bone fractures in subjects treated with exenatide but a reduced risk of nonvertebral fractures with liraglutide, as compared to other medications [97]. However, it is important to point that all the studies included in both meta-analyses were not specifically designed to evaluate fracture risk.

As for the effects of DPP-4 inhibitors on bone metabolism, no difference in bone fracture risk was observed between T2DM subjects treated with these drugs and nondiabetic controls, in a retrospective study using data from the Clinical Practice Research Datalink [98]. Conversely, a meta-analysis of 28 relatively short-term studies suggested that therapy with DPP-4 inhibitors is associated with a significant reduction in fracture risk, as compared to placebo or other antidiabetic agents [99].

### 5.6. Sodium Glucose Cotransporter 2 Inhibitors

Sodium glucose cotransporter 2 (SGLT2) inhibitors reduce glucose plasma levels by inhibiting proximal tubular reabsorption of glucose in the kidney. In addition to their demonstrated glycaemic efficacy, these drugs provide several clinical benefits, including body weight loss, the reduction of blood pressure values, and the low risk of hypoglycaemic events. Clinical data suggest that treatment with SGLT2 is associated with an increased risk of bone fractures. Because of their mechanism of action these drugs may influence calcium-phosphate homeostasis and potentially have an effect on bone metabolism and turnover. Several mechanisms may be involved: the raise of serum phosphate levels via increased tubular resorption of phosphate in the kidney; the increase of magnesium and PTH levels; the reduction of 25OH-vitamin D levels; and the increase of secretion of FGF23 by osteocytes; also weight loss frequently observed with SGLT2 may influence bone mass [100]. Serum phosphate, magnesium, and PTH levels are increased in subjects treated with dapagliflozin, as compared to those on placebo [101, 102]; however Ljunggren et al., in spite of a small increase in serum phosphate and magnesium levels, did not observe any changes from baseline in bone turnover markers or BMD after 50 weeks' treatment with dapagliflozin 10 mg/day versus placebo [101]. Furthermore, no risk of bone fracture associated with dapagliflozin emerged in a pooled analysis using data from 12 placebo-controlled studies [103]. Conversely, in a population of 252 T2DM subjects with moderate renal impairment treated with dapagliflozin or placebo for 104 weeks, low-trauma fractures were observed in 6% and 9.4% of patients on dapagliflozin 5 and 10 mg, respectively, whereas no bone fractures were reported in the placebo group [102].

Data on empagliflozin available to date do not show an increased risk of bone fracture or an impairment in BMD, compared to placebo [104].

Recently, a pooled analysis of nine clinical trials has shown that the use of canagliflozin is associated with an increased fracture incidence, particularly in women [105]. Furthermore a recent postmarketing safety study required by the Food and Drug Administration (FDA) showed a significant reduction in BMD at the lumbar spine and the hip, as detected by DXA, after 52 weeks of therapy with canagliflozin in a population of >700 elderly subjects [106]. Notably, the FDA has strengthened the warning for the increased risk of bone fractures with canagliflozin and invited health care professionals to consider factors that may contribute to this risk prior to starting treatment. The FDA revised the canagliflozin label and it is evaluating the risk of bone fractures with dapagliflozin and empagliflozin to determine if additional label changes or studies are needed. However effects of SGLT2 on bone fracture risk are not fully elucidated yet and further studies are needed to better clarify this issue. In particular it remains unclear if the effect on bone metabolism is a drug class-effect or there are differences between different molecules of the class, which may be sustained by differences in the degree of inhibition of renal cotransporter SGLT2 at maximum dosage, which is stronger for canagliflozin 300 mg than for dapagliflozin 10 mg.

## 6. Conclusions

Aging is associated with increasing prevalence of both diabetes and osteoporosis and these chronic diseases are frequently associated in the elderly, especially in women.

Although osteoporosis and T2DM seem to be unrelated from a pathophysiological standpoint, a number of epidemiological studies have demonstrated an increased fracture risk among patients with T2DM. This higher risk is likely due to a combination of greater risk of falling, regional osteopenia, and impaired bone quality and treatment effects.

Despite the well-documented higher fracture risk in T2DM, BMD measures show higher values in these patients. This apparent discrepancy is likely explained by the lower quality of bone in T2DM subjects, as documented by modern techniques investigating bone structure and strength, which may be more suitable to assess osteoporotic risk than DXA in these patients.

Different mechanisms have been proposed to explain the possible influences of diabetes on bone metabolism, including glycosuria, AGEs, low levels of IGF-I or alteration in plasma insulin levels, impaired kidney function, and chronic inflammation.

Also factors related to diabetes complications and/or to its management such as poor metabolic control or the use of some hypoglycaemic drugs may influence osteoporosis and/or fracture risk in T2DM patients. In particular, several medications used in the treatment of T2DM may have an impact on bone metabolism, and they should be used with caution in patients who are at risk for fall and/or fracture, particularly in postmenopausal T2DM women.

Thus, when considering the effects of hypoglycaemic drugs on bone it is important to balance their potential direct effects on bone metabolism with the risk of falling-related fractures due to the associated hypoglycaemic risk. Furthermore, for drugs which are usually prescribed in long-standing diabetes, such as insulin, the inclusion of subjects with diabetes micro- and macrovascular complications, especially retinopathy and neuropathy, should be taken into account when evaluating fracture risk.

Women are typically more exposed to osteoporosis risk, and men and women differ in terms of risk factors for falls and osteoporosis [107], but to date only few epidemiological studies specifically examined osteoporosis and fracture risk in T2DM men and women separately; furthermore, direct comparisons of men and women with T2DM are lacking. Although gender-related data on bone metabolism, bone measures, and fracture risk are too sparse to drew firm conclusions, available literature indicates that, because of diabetes, men may be less protected from osteoporosis than nondiabetic counterparts, although some authors reported no differences in BMD measures compared to nondiabetic men. Data on women are even more conflicting, with studies showing a higher BMD in those with than without T2DM. The inconsistency of literature on this issue may be related to the fact that BMD measures that have been extensively used to date are not the best marker of bone health in T2DM subjects, and more differences may emerge from future studies evaluating bone turnover markers in the two genders. As for hypoglycaemic drugs, thiazolidinediones are the only ones with well-documented negative effects on bone metabolism, which shows a gender dimorphism, being more clinically relevant in women than in men. This gender difference could be related to circulating estrogen levels, since estrogens reduce adipogenesis, the apoptosis of osteocytes, and the upregulation of sclerostin, which acts by inhibiting bone formation. However bone loss has been observed both in premenopausal and in postmenopausal women, so the matter remains unresolved. Unfortunately, little is known about the impact of most diabetes treatments on bone quality and specifically on fracture risk. Besides metformin and sulphonylureas, which do not seem to have specific effects on bone, several data point to a protective role of incretin-based therapies on fracture risk. These potential beneficial effects may rise from direct GLP-1R-mediated effects on bone metabolism but also from the low hypoglycaemic risk which may be protective against falling. Data on SGLT-2 inhibitors are still too sparse to identify whether the potential detrimental effects on bone are a class-effect or they are limited to specific drugs.

T2DM prevalence increases with age, and glucose lowering therapies are often prescribed in older subjects at higher fracture risk. Notably, although osteoporosis is typically a “female disease,” to date it is still largely unclear whether the bone effects of hypoglycaemic drugs are gender-specific. A better understanding of the real impact of diabetes treatments on bone quality and fracture risk is necessary to create personalized therapy, especially in subjects at higher risk, such as women and older patients.
