# Corrigendum to “Fracture Risk in Type 2 Diabetes: Current Perspectives and Gender Differences”

**DOI:** 10.1155/2017/4576102

**Published:** 2017-07-16

**Authors:** Giuseppina T. Russo, Annalisa Giandalia, Elisabetta L. Romeo, Nunziata Morabito, Marco Muscianisi, Maria Concetta Ruffo, Antonino Catalano, Domenico Cucinotta

**Affiliations:** Department of Clinical and Experimental Medicine, University of Messina, Messina, Italy

In the article titled “Fracture Risk in Type 2 Diabetes: Current Perspectives and Gender Differences” [1], with regard to the FRAX® tool, it has been reported that “Among the different tools to assess fracture risk, the WHO fracture risk assessment (FRAX) is a computer based algorithm (http://www.shef.ac.uk/FRAX/) primarily intended for use in primary care [56, 57].” We point out that the metabolic bone disease unit at the University of Sheffield that developed FRAX was a WHO Collaborating Centre from 1991 to 2010, but the World Health Organization (WHO) did not develop, test, or endorse the FRAX tool or its recommendations [2]. Additionally, the name of the fourth author was given incorrectly as Morabito Nunziata. The author's name should have been written as Nunziata Morabito. The revised authors' list is shown above.
